# The Salt Free Diet in Chronic Parenchymatous Nephritis

**Published:** 1907-04

**Authors:** 


### THE SALT FREE DIET IN CHRONIC PARENCHYMATOUS NEPHRITIS.

---

Peabody says that his experience is that in many cases very soon after the beginning of treatment for chronic parenchymatous nephritis, œdema, and therefore body weight will diminish rapidly. This occurs more promptly and more completely if the patient remains absolutely at rest in bed. In some cases œdema of the lower extremities does not entirely disappear, and in these diuretics must be employed. In general, the effect is much more marked in parenchymatous than in interstitial nephritis. The author has tried the effect of the salt free diet as a means of removing fluid in cases of anasarca of various kinds. In anasarca due to heart causes or to combined failure of heart power and interstitial nephritis, very little has been accomplished with it. Although the statement has been made that it causes a lowering of blood pressure in high tension with arteriosclerosis, such has not been the author's experience. The results of carefully conducted observations in cases of this class have been almost uniformly disappointing in his wards. The diet is not unpalatable, and can be made sufficiently varied to be well borne for weeks if necesary, though ordinarily a few days will suffice to remove the œdema, or greatly to diminish it. Unsalted bread is very palatable, especially if made with milk instead of water. Made in this way, it does not become hard or dry, if not too long kept. The following is the bill of fare adopted by the author: Breakfast: Coffee or tea, eggs, cereals, cream, fresh butter, fruits, bread made without salt. 10 a. m.: A glass of milk. Dinner: Chicken, fish, potato variously prepared, bread made without salt, ice cream, jelly, fresh butter cocoa 8 drachms. 3 p. m.: A glass of milk or water. Supper: Eggs, chicken, bread without salt, jelly, custard, cream, fresh butter, tea 8 drachms. 8 p. m.: A glass of milk or water. After complete removal of the œdema the author now administers progressively increasing daily quantities of salt to these patients, carefully watching for any recurrence of this symptom, the endeavor being to ascertain for each patient what his salt equilibrium may be. By far the best way of ascertaining the exact amount of water lost by such a patient is by weighing him. Measurement of the circumference of his abdomen and of his extremities is of little relative value. Unless some other illness supervenes, he loses no weight except water, for he is very well nourished by the food that he takes, so that by weighing him the author has a fairly exact means of ascertaining the result that we are bringing about. It is of interest that the disappearance of œdema is not always accompanied by any very obvious increase in the amount of urine passed, though this sometimes also happens. The patient must lose water in other ways, and he often loses both salt and water by the bowel.—Med. Record, March 9, 1907.

